# The relationship between three-dimensional knee MRI bone shape and total knee replacement—a case control study: data from the Osteoarthritis Initiative

**DOI:** 10.1093/rheumatology/kew191

**Published:** 2016-05-15

**Authors:** Andrew J. Barr, Bright Dube, Elizabeth M. A. Hensor, Sarah R. Kingsbury, George Peat, Mike A. Bowes, Linda D. Sharples, Philip G. Conaghan

**Affiliations:** ^1^Leeds Institute of Rheumatic and Musculoskeletal Medicine, University of Leeds and NIHR Leeds Musculoskeletal Biomedical Research Unit, Leeds, UK; ^2^Arthritis Research UK Primary Care Centre, Research Institute for Primary Care & Health Sciences, Keele University; ^3^Imorphics Ltd, Kilburn House, Manchester; ^4^Leeds Institute of Clinical Trials Research, University of Leeds, Leeds, UK

**Keywords:** osteoarthritis, knee, magnetic resonance imaging, 3D bone shape, active appearance modelling, total knee replacement

## Abstract

**Objective.** There is growing understanding of the importance of bone in OA. Our aim was to determine the relationship between 3D MRI bone shape and total knee replacement (TKR).

**Methods.** A nested case-control study within the Osteoarthritis Initiative cohort identified case knees with confirmed TKR for OA and controls that were matched using propensity scores. Active appearance modelling quantification of the bone shape of all knee bones identified vectors between knees having or not having OA. Vectors were scaled such that −1 and +1 represented the mean non-OA and mean OA shapes.

**Results.** Compared to controls (n = 310), TKR cases (n = 310) had a more positive mean baseline 3D bone shape vector, indicating more advanced structural OA, for the femur [mean 0.98 *vs* −0.11; difference (95% CI) 1.10 (0.88, 1.31)], tibia [mean 0.86 *vs* −0.07; difference (95% CI) 0.94 (0.72, 1.16)] and patella [mean 0.95 *vs* 0.03; difference (95% CI) 0.92 (0.65, 1.20)]. Odds ratios (95% CI) for TKR per normalized unit of 3D bone shape vector for the femur, tibia and patella were: 1.85 (1.59, 2.16), 1.64 (1.42, 1.89) and 1.36 (1.22, 1.50), respectively, all P < 0.001. After including Kellgren–Lawrence grade in a multivariable analysis, only the femur 3D shape vector remained significantly associated with TKR [odds ratio 1.24 (1.02, 1.51)].

**Conclusion.** 3D bone shape was associated with the endpoint of this study, TKR, with femoral shape being most associated. This study contributes to the validation of quantitative MRI bone biomarkers for OA structure-modification trials.

Rheumatology key messages

- Three-dimensional MRI-assessed bone shape in OA is associated with subsequent total knee replacement.
- Bone represents an increasingly validated biomarker for assessing OA progression in clinical trials.

## Introduction

OA is one of the most common causes of global disability [1] and its prevalence is expected to increase as people live longer and become more obese [2–4]. While there are no licensed disease-modifying agents in OA, the unmet need for such therapies is huge. In order to demonstrate the efficacy of structural modification, biomarkers that accurately measure structural progression are required. Until recently, joint space narrowing seen on conventional radiography has been the surrogate measure of hyaline cartilage loss or structural progression. However this is less sensitive and specific in detecting structural pathology and structural progression than MRI [5, 6]. MRI has established that OA is a whole-joint disease and typically affects all of the tissues within the joint. MRI also identifies early structural pathology that is present in the asymptomatic pre-radiographic stages of knee OA [5] and is associated with incident symptomatic [7] and radiographic disease [8]. The 3D data incorporated in MRI permit quantification of tissue pathology through manual segmentation [9–12], or automated analysis techniques such as active appearance modelling (AAM). These automated methods facilitate rapid and accurate quantification of large datasets [13–15].

Subchondral bone is integral to the pathogenesis and progression of OA [16, 17]. While much of the relevant OA literature has focused on MRI-detected bone marrow lesions, there is a growing literature on the importance of bone shape [8, 15, 18, 19]. In particular, the expansion of 3D bone surface area at the femoro-tibial articulation is larger in OA knees than healthy controls and correlates with knee joint space narrowing, osteophytes and Kellgren–Lawrence (KL) grade after adjusting for appropriate confounders in cross-sectional studies [9, 10, 12, 20]. Subchondral bone surface area is also strongly associated with structural progression [21–24]. As we develop more understanding of the bone in OA as a potential biomarker, we need to understand its relationship to important patient outcomes such as total knee replacement (TKR). The objective of this nested case–control analysis was to determine the relationship between 3D bone shape and TKR.

## Methods

Data used for this nested case-based, case–control analysis, with cumulative incidence sampling, were obtained from the NIH Osteoarthritis Initiative (OAI) database, which is available for public access at http://www.oai.ucsf.edu/. This database houses a multicentre, prospective, longitudinal observational study of knee OA currently including approximately 4796 participants [25]. Baseline demographic data were collected and MRI scans were performed for all participants. At each site the Institutional Review Board (IRB) approved the study and informed consent was given by all patients. The OAI study and public use of clinical and imaging data used in our study was approved by the committee on Human Research, University of California, San Francisco (IRB approval number 10-00532).

For the current study, definition of case knee or control knee status required baseline records of age, gender, knee numeric pain scale, weight and KL grade. A prerequisite for eligibility also included having a baseline MRI knee scan and confirmed knee replacement status by the 72 month follow-up visit. Cases were defined as any knee with the following: confirmed TKR status (patient-reported TKR and adjudicated confirmed status on subsequent radiograph between baseline and the 72 month follow-up visit); confirmed OA indication for TKR or, where this was unrecorded, an OA indication was highly likely (the presence of baseline radiographic and symptomatic evidence of OA) for the replaced knee. Where a participant had both knees replaced during the follow-up (n = 69), the first knee to be replaced was included (n = 50). Where both knees were replaced on the same day (n = 19) one knee was randomly selected. Knees that survived were eligible for selection as controls if they had neither patient-reported nor post-knee replacement adjudicated confirmation on follow-up radiograph at the 6 year follow up point. The OAI cohort has excellent follow-up retention rates at 6 years (∼88%). Where two control knees were available for the same participant, the knee with the highest pain score, or the right side if they reported equal pain, was selected. Control knees were matched 1:1 with case knees using propensity score (PS) matching (described below). With multiple strong confounders (e.g. age, gender and BMI), PS matching was chosen as an efficient way of creating an unbiased comparison and improving precision.

MRI sequences collected in the OAI are described in detail by Peterfy and colleagues [26]. The current study utilized the double-echo-in-steady-state water excitation sequence of the Siemens 3T trio systems [26].

The quantitative analysis of 3D bone shape was achieved by automated segmentation of the 3D MRI double-echo steady-state water excitation sequences. AAMs are statistical shape modelling methods that learn the variation in shape and grey-scale texture (appearance) of objects from a training set (such as the bones from an MRI), and encode shape and appearance as principal components [13, 14].

The AAM methodology has been previously published [8]. In summary a training set of 96 knees with equal numbers of knees from each KL grade was used to build AAMs for each knee bone so that the trained AAMs can automatically segment bones in MR images. The validation of the accuracy of the knee segmentations has been previously described [14, 27]. For our particular models, the accuracy of the automated segmentations was further assessed using test–retest MRIs for 19 participants (38 images) with no OA to moderate degrees of clinical OA, prepared as a pilot study for the OAI, using the same MRI sequence [28]. The bone surface was manually segmented as previously described [29]. Mean point-to-surface distances were calculated between the manual and automated segmentations. Mean point-to-surface errors were as follows: for the femur, 0.49 mm; for the tibia, 0.53 mm; and for the patella, 0.57 mm (i.e. each approximately the size of 1 voxel) [8]. The reliability of the automated segmentations has been established [28, 29].

While segmentation for cartilage thickness has a coefficient of the variance of over 2%, bone segmentation has a coefficient of the variance of 0.8–1.9% [29]

In order to identify vectors within this shape space that represented average change of the bone with disease, we separated the 96 training set cases into OA (KL grade 2–4, n = 53) and non-OA groups (KL 0–1, n = 43)

**Fig. 1:**
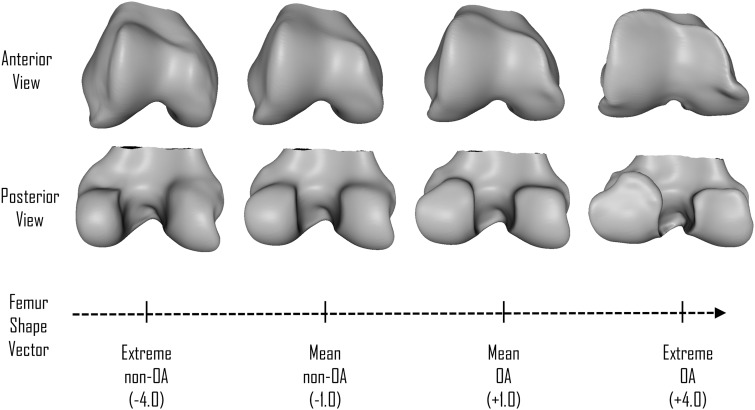
Scalar continuous vector of 3D bone shape of the femur Anterior and posterior views of right knees. The femoral shape vector is scaled to –1 as the mean shape without radiographic OA and +1 with established radiographic OA.

The shape vector for each bone is calculated by taking the principal components of the mean shape of the OA and non-OA groups, and drawing a straight line through them. Individual bone shapes from knees in this study, represented as principal components following the AAM search, were projected orthogonally onto the vector. The distance along the vector was normalized by treating the mean non-OA shape as −1 and the mean OA shape as +1 (Fig. 1).

The reproducibility or test–retest reliability of the method was described in a set of 35 OA knees that underwent MRI 1 week apart at one single OAI site, using the same OAI image acquisition sequence [29]. The reproducibility for the OA vector was good, with the smallest detectable difference (95% confidence limits on a Bland–Altman plot) being 0.3 (1%) normalized units for the whole joint model [8]. The smallest detectable difference for the femur, tibia and patella bone shape vectors was 0.22, 0.86 and 0.24 units normalized to the mean non-OA shape, respectively [30]. 

### Statistical analysis

Statistical analysis was conducted using Stata software, version 13 (2013; StataCorp, College Station, TX, USA). Matching of control knees to case knees 1:1 was performed using PS matching. The PS is the conditional probability of assignment to a particular treatment given a vector of covariates and has been shown to be sufficient in removing bias due to observed covariates in observational studies [31]. In this analysis the PS is the conditional probability of receiving a TKR given a vector of covariates. Our model was based on the probability of having the outcome of TKR conditional on observed covariates. This is stratification score matching, which has been previously described [32] and is referred to as PS matching in this retrospective analysis. The propensity model was estimated using logistic regression based on *a priori* known relationships between the outcome TKR and clinically important risk factors such as age, gender, weight, KL grade and pain [33], which may influence the surgeon’s decision to operate. We considered whether health insurance could affect the outcome with participants potentially not offered a TKR for financial reasons; however on exploration of the data we found that 98% of participants that had a TKR had some form of health insurance while 96% of those not having a TKR had insurance.

The final logistic regression model included baseline age, gender, weight category, ipsilateral knee pain severity numeric rating scale (range 0–10), and knee side (right or left) (supplementary Table S1, available at *Rheumatology* Online). KL grade was deliberately omitted because it directly influences the decision for TKR by the orthopaedic surgeon, resulting in over-matching. Furthermore the KL grade is used to define the scalar 3D bone shape variable and hence the two are moderately correlated (Barr *et al.*, unpublished data).

Age was categorized into 5-year groupings prior to inclusion in the PS model. Weight was categorized using WHO BMI categories (kg/m^2^): underweight (<18.5), normal (18.5–24.9), overweight (25–29.9) and obese (⩾30). Underweight and normal categories were subsequently merged because very few patients were underweight (n = 10). Control knees were matched to case knees with the nearest PS (nearest neighbour matching) without replacement. Matching between case and control knees was confirmed graphically and by comparing the means or medians for the PS and the distribution of the components of the PS between case and control knees. Age, BMI and pain were analysed as continuous variables and gender kept as dichotomous. The variables were considered well balanced if the standardized difference percentage (the mean difference as a percentage of the average standard deviation) for continuous variables was <10% as values greater than this are reported to represent meaningful imbalance [34], and for dichotomous variables a standardized difference (difference between prevalence in treatment and control group divided by an estimate of within-group standard deviation) <0.1 represented good balance [35].

For simple comparison between the matched groups, Student’s t-tests for paired samples were used to compare 3D bone shapes of the femur, tibia and patella between case and control knees. Univariable and multivariable conditional logistic regression analyses were performed to establish the association of each baseline 3D bone shape vector as continuous variables with the outcome of TKR. Multivariable analyses included KL grade, and the Akaike information criterion (AIC) was used to compare models with and without 3D bone shape, in order to assess any additional association beyond that between KL grade and TKR. Model fit was assessed by examining leverage plots to identify pairs that did not fit the data well, and also observations exerting a strong influence on the estimated coefficients. The model fitted the data well based on diagnostic checks. We also analysed 3D bone shape vectors categorized into tertiles with the highest tertile containing values closer to mean OA shape and the lowest tertile containing values closer to the mean non-OA shape.

Additional exploratory analyses (conditional logistic regression models) aimed to determine whether any association of bone shape with TKR was maintained over all KL grades and to establish if there were any differences in association between 3D bone shape and TKR depending on the time from baseline to TKR date in years.

## Results

Of the 4796 participants in the OAI database, 336 individuals with at least one confirmed knee replacement during follow-up were identified. In total 405 knees had been replaced (right 198, left 207). Of the 69 patients who had both knees replaced, 50 patients’ knees were replaced on separate days, and from them 25 right knees and 25 left knees were selected for analysis on the basis that they were replaced first. From the remaining 19 patients whose knees were replaced on the same day, 11 right knees and 8 left knees were randomly selected. Only one knee was excluded by TKR indication that was recorded as RA. Having excluded patients with missing MRI data, 310 case knees met our criteria and were suitable for PS matching (supplementary Fig. S1, available at *Rheumatology* Online).

The 336 individuals with knee replacements differed from the rest of the OAI cohort in baseline mean age (3 years older), KL grades, obesity and numeric rating scale pain scores (Table 1).

**Table 1:** Characteristics of participants according to the presence or absence of a total knee replacement

Clinical and demographic variables	TKR (n = 336)	No TKR (n = 4460)
Age, mean (s.d.), years	64.1 (8.39)	60.8 (9.19)
Gender, female, %	60	58
Health insurance, %	98	96
Weight, n (%)		
Normal/underweight	44 (13)	2004 (25)
Overweight	131 (39)	1746 (39)
Obese	161 (48)	1610(36)
KL grade, n (%)		
0	11 (3)	1560 (35)
1	15 (5)	715 (16)
2	74 (22)	1112 (25)
3	136 (41)	591 (13)
4	94 (28)	157 (4)
Missing	6 (1)	325 (7)
NRS, median (IQR)	5 (3–7)	3 (1–5)

The characteristics of 4796 participants of the OAI according to the presence or absence of at least one confirmed, adjudicated total knee replacement before matching. IQR: interquartile range; KL: Kellgren–Lawrence; NRS: Numerical Rating Scale for pain; TKR: total knee replacement.

The number of exact PS matches was 244 (79%) with all remaining case knees having a PS within 0.01 PS units of their control knee. A minority of case knees had been matched with contralateral control knees [n = 12 (4%)]. There was a concordant gender match in all case–control knee pairs.

There were no substantive differences between the cases and matched controls (Table 2). The distribution of the PSs in the two groups was similar (supplementary Fig. S2, available at *Rheumatology* Online). The standardized differences for age, BMI and Numerical Rating Scale pain were 3.3, 0.5 and 1.8%, respectively (all within 10%), while gender was matched perfectly.

**Table 2:** Demographic characteristics of participants with knee replacement and their controls from propensity matching

Clinical and demographic variables	TKR cases	Controls
	(n = 310)	(n = 310)
Gender, male, n (%)	123 (40)	123 (40)
Age categories, n (%)		
45–49	12 (4)	17 (5)
50–54	34 (11)	35 (11)
55–59	53 (17)	45 (15)
60–64	53 (17)	59 (19)
65–69	64 (21)	59 (19)
70–74	52 (17)	56 (18)
75+	42 (13)	39 (13)
Weight category, n (%)		
Normal/underweight	165 (53)	157 (51)
Overweight	156 (47)	153 (49)
Health insurance^a^, n (%)	301 (98)	292 (95)
Side, right, n (%)	159 (51)	161 (52)
NRS, median (IQR)		
Left knee score	5 (3–7)	5 (3–6)
Right knee score	5 (2–7)	5 (3–7)
KL grade^a^, n (%)		
0	10 (3)	93 (30)
1	12 (4)	49 (16)
2	69 (22)	85 (27)
3	128 (42)	47 (15)
4	85 (27)	13 (4)
Missing	6 (2)	23 (7)

^a^Not used in propensity matching. IQR: interquartile range; KL: Kellgren–Lawrence; NRS: Numerical Rating Scale for pain; TKR: total knee replacement.

The mean baseline 3D bone shape scalar variable indicated significantly greater structural severity in cases of TKR than in controls. Mean (s.d.) in the femur cases was 0.98 (1.51) compared with controls mean of −0.11 (1.40), with a statistically significant difference of 1.10 (95% CI: 0.88, 1.31), t (309) = 10.31, P < 0.001. Mean (s.d.) in the patella cases was 0.95 (1.84) compared with a control mean of 0.03 (1.83), with a statistically significant difference of 0.92 (95% CI: 0.65, 1.20), t (309) = 6.61, P < 0.001. The femur had the largest mean difference in 3D shape of the three knee bones (Table 3).

**Table 3:** The mean differences between bone shape vectors of cases and controls

	Bone vector (mean (s.d.))			
Bone	Control	TKR	Mean difference (95% CI)	P-value^a^
Femur	–0.11 (1.40)	0.98 (1.51)	1.10 (0.88, 1.31)	<0.001
Tibia	–0.07 (1.39)	0.86 (1.42)	0.94 (0.72, 1.16)	<0.001
Patella	+0.03 (1.83)	0.95 (1.84)	0.92 (0.65, 1.20)	<0.001

^a^Paired t-test.

The conditional logistic regression models of each individual bone estimated increased odds of TKR with more positive 3D bone shape, indicating greater OA structural severity (Table 4). Odds ratios (ORs) (95% CIs) per normalized unit of 3D bone shape vector for the femur, tibia and patella were 1.85 (1.59, 2.16), 1.64 (1.42, 1.89) and 1.36 (1.22, 1.50), respectively, all P < 0.001. The order of strength of association of bone shape with TKR, from strongest to weakest, was therefore the femur, the tibia and finally the patella. The odds on TKR increased significantly with KL grade.

**Table 4:** The associations between 3D bone shape vectors or KL grade with TKR

	
	Univariable (unadjusted)							Multivariable^a^		
Imaging variable	OR (95% CI)			P-value		AIC		OR (95% CI)	P-value	AIC
Femur vector	1.85 (1.59, 2.16)			<0.001		341.11		1.2 (1.02, 1.51)	0.03	228.33
Tibia vector	1.64 (1.42, 1.89)			<0.001		367.80		1.09 (0.90, 1.32)	0.40	232.66
Patella vector	1.36 (1.22, 1.50)			<0.001		390.84		1.06 (0.92, 1.23)	0.40	231.24
Combined bone vectors										
Femur vector								1.26 (1.00, 1.60)	0.05	
Tibia vector								0.97 (0.78, 1.21)	0.80	
Patella vector								1.00 (0.85, 1.17)	0.96	235.00
KL grade (ref = KL zero)										
1	1.66 (0.58, 4.73)			0.34						
2	5.66 (2.55, 12.55)			<0.001						
3	17.18 (7.43, 39.72)			<0.001						
4	39.77 (14.64,108.0)			<0.001		230.70				

^a^Adjusted for KL grade. AIC: Akaike’s information criterion; KL: Kellgren–Lawrence; OR: Odds ratio from conditional logistic regression; TKR: total knee replacement.

When KL was included in multivariable conditional logistic regression models, the femur remained significantly associated with TKR but the association with the tibia and patella was attenuated. The ORs (95% CIs) per normalized unit of 3D bone shape vector were 1.24 (1.02, 1.51), 1.09 (0.90, 1.32) and 1.06 (0.92, 1.23) for the femur, tibia and patella, respectively. The AIC of the univariable KL model (230.70) was lower than any of the univariable bone vector models and multivariable tibia and patella models and the strength of association was in the expected direction (OR for KL3 was greater than that of KL2 when compared with KL0 as reference). For the adjusted models, based on AIC, the adjusted femur model was the best model and favoured over the more complex model with all three vectors included together (Table 4). Those in the highest tertile for the femur bone shape vector had 12.7 times higher chance than those in the lowest tertile group of having a TKR (95% CI: 6.93, 23.40, P < 0.001). In all analyses a similar trend was noted with those in the highest tertile having higher chances of having a TKR than both the middle tertile and lowest tertile. After adjusting for KL grade, the highest tertile of the femur vector was most significantly associated with TKR (Table 5).

**Table 5:** The associations between 3D bone shape vectors TKR using lowest tertile as reference

3D bone shape vector	OR (95% CI)	P-value	Adjusted OR (95% CI)^a^	P-value
Femur				
Highest tertile	12.73 (6.93, 23.40)	<0.001	3.47 (1.62, 7.47)	0.001
Middle tertile	2.55 (1.60, 4.05)	<0.001	1.17 (0.65, 2.08)	0.610
Lowest tertile (reference)	1.0			
Tibia				
Highest tertile	6.32 (3.84, 10.40)	<0.001	1.95 (0.99, 3.82)	0.052
Middle tertile	2.80 (1.72, 4.56)	<0.001	1.64 (0.85, 3.15)	0.140
Lowest tertile (reference)	1.0			
Patella				
Highest tertile	3.81 (2.40, 6.08)	<0.001	1.78 (0.92, 3.44)	0.088
Middle tertile	2.26 (1.47, 3.49)	<0.001	1.84 (1.01, 3.35)	0.046
Lowest tertile (reference)	1.0			

^a^Adjusted for KL grade. KL: Kellgren–Lawrence; TKR: total knee replacement.

The association between bone shape and TKR was not significantly modified when stratified by KL grade severity (supplementary Tables S2–S4, available at *Rheumatology* Online). Similarly the association between bone shape and TKR was not significantly modified by the length of the interval between baseline and TKR incidence (supplementary Tables S5 and S6, available at *Rheumatology* Online). However these were exploratory analyses that included small numbers in each stratum with large confidence intervals. The cumulative incidence of the 310 TKR cases over ∼7 years is relatively stable over the first 7 years (supplementary Table S5, available at *Rheumatology* Online).

## Discussion

This is the first study to describe the association between 3D bone shape and TKR. We demonstrated that people having TKR exhibited significantly more advanced 3D shape changes of OA structural progression than controls at baseline. TKR has been previously associated with age, obesity, pain characteristics, radiographic OA severity and MRI features such as cartilage damage [36–40]. MRI-determined bone shape is strongly related to OA structural progression [15]. The association of OA subchondral bone pathology with TKR is supported by the association of bone marrow lesions with TKR, though these are likely to be measuring a different pathological construct [37–39].

The OR of TKR per normalized unit of 3D bone shape vector increased with greater baseline 3D shape structural severity for each of the femur, tibia and patella. However femoral bone shape has the strongest association with TKR. It is worth noting that the femoral bone shape had the largest scalar value difference between case and control knees and is the only bone shape that, after adjusting for KL grade, remained significantly associated with the outcome of TKR. These findings concur with the same 3D femur shape having the largest hazard ratio of the three knee bones in predicting incident radiographic knee OA [18]. Furthermore the 3D bone area of femoral trochlear and tibio-femoral articulations were the most responsive and had the greatest percentage change in size of all articulating surfaces in the knee [15]. Cartilage loss from the femur was better than loss from the tibia in distinguishing OA knees requiring and not requiring knee replacement in a previous study [33]. The tibial 3D shape changes in a more uniform and symmetric pattern than the femur where the shape change distinctly occurs around the cartilage plates where an increased ridge of osteophytic material forms [8]. This may explain why femoral 3D shape has a greater responsiveness and association with TKR. While the shape of the bone readily changes in response to mechanical forces acting upon it (Wolff’s law) [19, 41], it is reasonable that the femur shape should change more than that of the tibia or patella because the femur receives more load from twice the number of weight-bearing surfaces as the tibia and patella.

The association of TKR with the highest tertile of femoral bone shape persists after adjusting for KL grade, but the association of TKR with the highest tertiles of the tibial and patellar bone shape are attenuated (Table 5). This may reflect the observation that in more advanced structural progression, tibial and patellar shape change is more uniform and symmetrical than that of the femur [8, 15]. This more readily detectable shape change in the femur may explain the stronger association with TKR in more advanced structural progression (the higher tertiles). The additional structural information provided by 3D bone imaging compared with conventional radiography [20] may explain why in the exploratory analyses the association with TKR was maintained after stratifying for KL grade severity, indicating the stage of structural severity does not affect this association.

The requirements of biomarker validation have been described [42]. The findings of this study in conjunction with previous data on construct validity, reliability and responsiveness of 3D bone shape indicate that novel quantitative measures of bone shape are increasingly validated biomarkers of OA structural progression [8, 15, 18]. 3D bone shape has advantages over conventional ordinal and metric radiographic measures of structural progression in that it provides a continuous measurement variable that is a more responsive measure in radiographic knee OA [15] and is sensitive to pre-radiographic knee OA [8].

There are limitations to this study. Joint arthroplasty is an end-point reflecting symptom and structural damage severity, but many variables influence both the timing and the decision to perform this outcome measure. The surgeon’s opinion and the patient’s comorbidities and willingness to undergo surgery are examples of these, in addition to local and national health system variations in surgical waiting lists that may cause residual confounding.

There is no consensus on which variables to include in the propensity model with studies showing a similar effect if all measured baseline covariates, all potential confounders or all true confounders are used interchangeably [35]. It has, however, been demonstrated that in order to maximize the number of matched pairs, the propensity model specified should have only the true or potential confounders [34]. In our sample 79% of case and control knees were matched exactly. This PS method adjusted for imbalances at baseline but not over time. Body mass index may increase before TKR and this may cause residual confounding conceivably with greater BMI increases in the TKR cases than in the controls. This may influence the size of the associations described in this analysis.

An alternative study design considered was cox-regression analysis, modelling the time-to-event (in this case TKR), which could have compared different survival times based on the 3D bone vector as well as estimates of covariates using the full cohort. However, since our objectives did not include estimating the effect of these well described and strong predictors of TKR, and knee replacement satisfied the rare outcome assumption (prevalence of 7%), we chose to perform a well-matched case-control analysis instead of a whole cohort survival analysis. Essebag and colleagues [43] highlighted this relative advantage of well-matched case–control analysis where the effect of the confounding factors is not of interest. A case–control analysis therefore represented a more precise analysis by efficient matching of multiple strong confounding factors to minimize the effect of bias.

We also acknowledge that by effectively matching cases and controls we may inadvertently overmatch, which may reduce any effect of 3D bone shape on the outcome of TKR, and our chosen method is therefore at risk of underestimating the magnitude of the association of bone shape vector with the outcome of TKR.

Our multivariable analyses are potentially subject to the effects of multiple collinearity. This is because KL grade is moderately correlated with bone shape vectors and the three bone shape vectors from within the same knee are used in the same multivariable models.

The OAI is predominantly a Caucasian cohort with smaller numbers of other ethnic groups. This means that any attempt to match cases and controls based on ethnicity would limit the available pool for matching. We acknowledge that by not matching on ethnicity there may be residual confounding although race was not associated with TKR incidence after covariate adjustment in the OAI dataset [40]. Health insurance was available for 98% of participants in our study suggesting any differences in ethnicity between cases and controls did not reflect their access to TKR surgery. The associations reported in this analysis should not be generalized to non-Caucasian populations due to the lower representation of non-White participants in this analysis. Additionally, as with any observational study, it is impossible to rule out residual bias from unknown or unmeasured confounders.

Magnetic resonance cannot directly identify the presence of calcium. In the segmentation of knee, double-echo-in-steady-state water excitation MRI sequences the material imaged is assumed to represent bone rather than another tissue type. Confirmation that these surfaces are actually bone requires further work. Finally the automated segmentation used here is both accurate and repeatable; however, some subtle details of particular diseases may not have been identified [44, 45].

In conclusion, 3D bone shape was associated with the important patient outcome of TKR and the femur had the greatest association. The predictive validity demonstrated here further underpins the value of quantitative bone measures in future therapeutic trials of disease-modifying OA drugs.

Author contributions: A.J.B., B.D., E.M.A.H., S.R.K., G.P., M.A.B., L.D.S. and P.G.C. contributed to the planning and design of this analysis. A.J.B. and B.D. drafted the article and E.M.A.H., S.R.K., G.P., M.A.B., L.D.S. and P.G.C., revised the article. All authors approved the final version for publication.

## Supplementary Material

Supplementary Data
