# Supplementary material for: The relationship between three-dimensional knee MRI bone shape and total knee replacement—a case control study: data from the Osteoarthritis Initiative
Source: Rheumatology (Oxford). 2016 May 15;55(9):1585–93. doi: 10.1093/rheumatology/kew191 (PMC4993955; doi:10.1093/rheumatology/kew191)
Supplement: Supplementary Data [file supp_55_9_1585__index.html]

The relationship between three-dimensional knee MRI bone shape and total knee replacement—a case control study: data from the Osteoarthritis Initiative — The relationship between three-dimensional knee MRI bone shape and total knee replacement—a case control study: data from the Osteoarthritis Initiative — Supplementary Data 

# The relationship between three-dimensional knee MRI bone shape and total knee replacement—a case control study: data from the Osteoarthritis Initiative

## Supplementary Data

files

- Supplementary Data - docx file
