# Supplementary material for: The relationship between three-dimensional knee MRI bone shape and total knee replacement—a case control study: data from the Osteoarthritis Initiative
Source: Rheumatology (Oxford). 2016 May 15;55(9):1585–93. doi: 10.1093/rheumatology/kew191 (PMC4993955; doi:10.1093/rheumatology/kew191)
Supplement: Supplementary Data [file supp_kew191_rhe-15-0530-File003.docx]

# Supplementary DATA

## Supplementary Figure S1. Participant flow diagram for the case selection


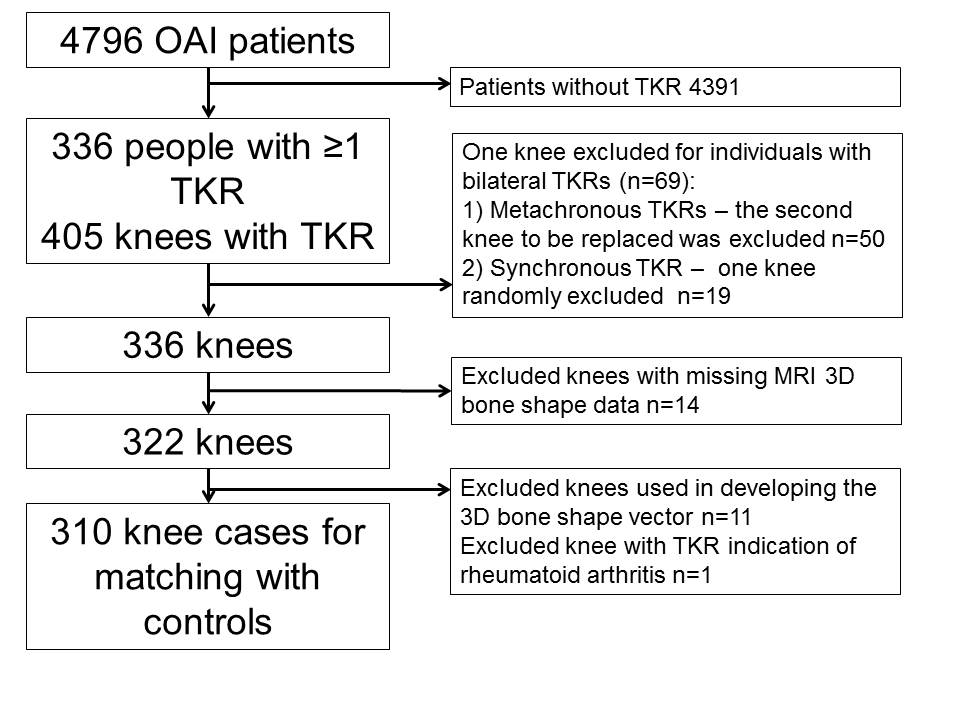


OAI: Osteoarthritis Initiative; TKR: Total Knee Replacement.

## Supplementary Figure S2. The distribution of propensity scores amongst cases and controls


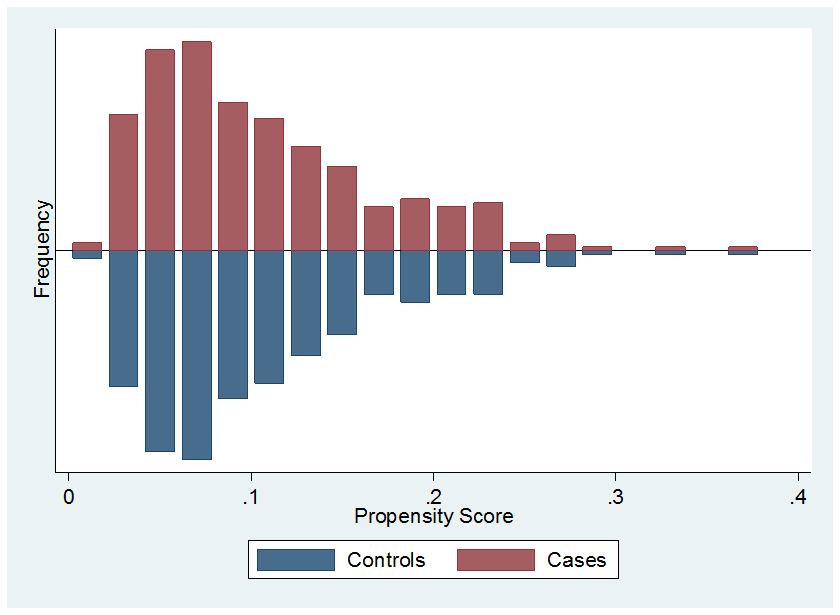


## Supplementary Table S1. Results from propensity model used to match cases and controls

| Outcome -TKR | Coefficient | 95% CI | P value |
| --- | --- | --- | --- |
| Age category (ref 0) |  |  |  |
| 1 | 0.58 | -0.05, 1.22 | 0.072 |
| 2 | 1.15 | 0.55, 1.76 | <0.001 |
| 3 | 1.28 | 0.68, 1.88 | <0.001 |
| 4 | 1.60 | 1.00, 2.19 | <0.001 |
| 5 | 1.47 | 0.87, 2.08 | <0.001 |
| 6 | 1.76 | 1.13, 2.39 | <0.001 |
| Sex (ref male) |  |  |  |
| Female | -0.00027 | -0.24, 0.24 | 0.99 |
| NRS | 0.17 | 0.13, 0.21 | <0.001 |
| Weight category(ref normal) |  |  |  |
| Overweight | 0.38 | 0.14, 0.62 | 0.002 |
| Side (ref Right) | 0.60 | 0.36, 0.84 | <0.001 |
| Constant | -4.87 | -5.47, -4.27 | <0.001 |

NRS: Numerical Rating Scale for pain; TKR: Total Knee Replacement.

## Supplementary Table S2. The number of propensity score matched pairs of cases and controls with equal KL knee grades

| Kellgren Lawrence grade | Pairs of knees | Number of knees |
| --- | --- | --- |
| 0 | 2 | 4 |
| 1 | 4 | 8 |
| 2 | 14 | 28 |
| 3 | 18 | 36 |
| 4 | 3 | 6 |
| Total | 41 | 82 |

KL: Kellgren Lawrence grade.

## Supplementary Table S3. The number of propensity score matched pairs of cases and controls with equal KL grades

| Kellgren Lawrence grade | Pairs of knees | Number of knees |
| --- | --- | --- |
| 0,1 | 6 | 12 |
| 2 | 14 | 28 |
| 3,4 | 21 | 42 |
| Total | 41 | 82 |

The number of propensity score matched pairs of cases and controls with equal KL knee grades after grouping into KL grade strata. KL: Kellgren Lawrence grade.

**Supplementary Table S4. The difference in mean bone shape vector between propensity score matched cases and controls**

| Bone | Kellgren Lawrence grade | Mean bone shape vector difference between vectors matched cases and controls with identical KL grade | Standard deviation | Confidence interval | Effect size^a^ |
| --- | --- | --- | --- | --- | --- |
| Femur | 0,1 | 0.78 | 1.56 | -0.85, 2.42 | 0.50 |
|  | 2 | 0.66 | 1.55 | -0.23, 1.56 | 0.43 |
|  | 3,4 | 0.12 | 2.45 | -0.99, 1.23 | 0.05 |
| Tibia | 0,1 | 0.45 | 0.99 | -0.58, 1.50 | 0.46 |
|  | 2 | 0.59 | 1.39 | -0.21, 1.39 | 0.42 |
|  | 3,4 | 0.38 | 2.17 | -0.61, 1.37 | 0.18 |
| Patella | 0,1 | 0.07 | 1.43 | -1.43, 1.57 | 0.05 |
|  | 2 | 0.74 | 2.77 | -0.86, 2.35 | 0.27 |
|  | 3,4 | 0.32 | 2.66 | -0.89, 1.53 | 0.12 |

The difference in mean bone shape vector between propensity score matched cases and controls with equal KL grades after division into strata. The association between bone shape and TKR was not significantly modified by KL grade severity strata. However this is an exploratory analysis with large confidence intervals, using small numbers of matched case control pairs. ^a^The mean bone shape vector difference (column 3) divided by the standard deviation (column 4). TRK: Total Knee Replacement; KL: Kellgren Lawrence grade.

## Supplementary Table S5. The cumulative incidence of the TKR cases used for the case-control analysis

| Year of case TKR | Incidence of case TKRs | Cumulative Incidence of case TKRs |
| --- | --- | --- |
| 0-1 | 23 | 23 |
| 1-2 | 37 | 60 |
| 2-3 | 39 | 99 |
| 3-4 | 52 | 151 |
| 4-5 | 54 | 205 |
| 5-6 | 48 | 253 |
| 6-7 | 51 | 304 |
| 7-8 | 6 | 310 |
| Total | 310 | 310 |

TKR: Total Knee Replacement

## Supplementary Table S6. The association of 3D femur bone shape vector with TKR by annual TKR incidence

| The year of incidence of case TKR | OR | 95% CI | P value |
| --- | --- | --- | --- |
| All years combined | 1.85 | 1.59, 2.16 | <0.001 |
| 0 to 1 | 1.92 | 1.17, 3.18 | 0.010 |
| 1 to 2 | 1.85 | 1.20, 2.87 | 0.006 |
| 2 to 3 | 2.68 | 1.44, 4.99 | 0.002 |
| 3 to 4 | 1.57 | 1.12, 2.18 | 0.008 |
| 4 to 5 | 1.82 | 1.25, 2.66 | 0.002 |
| 5 to 6 | 2.30 | 1.39, 3.81 | 0.001 |
| 6 to 8 | 1.60 | 1.15, 2.24 | 0.006 |

The association between bone shape and TKR was not significantly modified by the time of TKR from baseline. However this is an exploratory analysis with large confidence intervals, using small numbers of matched case control pairs. TKR: Total Knee Replacement.
